# A cross population between *D*. *kaki* and *D*. *virginiana* shows high variability for saline tolerance and improved salt stress tolerance

**DOI:** 10.1371/journal.pone.0229023

**Published:** 2020-02-25

**Authors:** Francisco Gil-Muñoz, Juan Gabriel Pérez-Pérez, Ana Quiñones, Amparo Primo-Capella, Jaime Cebolla, Mª Ángeles Forner-Giner, Maria L. Badenes, Mª del Mar Naval

**Affiliations:** 1 Instituto Valenciano de Investigaciones Agrarias (IVIA), Moncada, Valencia, Spain; 2 Instituto de Conservación y Mejora de la Agrodiversidad Valenciana (COMAV), Camino de Vera, Valencia, Spain; University College Dublin, IRELAND

## Abstract

Persimmon (*Diospyros kaki* Thunb.) production is facing important problems related to climate change in the Mediterranean areas. One of them is soil salinization caused by the decrease and change of the rainfall distribution. In this context, there is a need to develop cultivars adapted to the increasingly challenging soil conditions. In this study, a backcross between (*D*. *kaki* x *D*. *virginiana*) x D. kaki was conducted, to unravel the mechanism involved in salinity tolerance of persimmon. The backcross involved the two species most used as rootstock for persimmon production. Both species are clearly distinct in their level of tolerance to salinity. Variables related to growth, leaf gas exchange, leaf water relations and content of nutrients were significantly affected by saline stress in the backcross population. Water flow regulation appears as a mechanism of salt tolerance in persimmon via differences in water potential and transpiration rate, which reduces ion entrance in the plant. Genetic expression of eight putative orthologous genes involved in different mechanisms leading to salt tolerance was analyzed. Differences in expression levels among populations under saline or control treatment were found. The ‘High affinity potassium transporter’ (*HKT1-like*) reduced its expression levels in the roots in all studied populations. Results obtained allowed selection of tolerant rootstocks genotypes and describe the hypothesis about the mechanisms involved in salt tolerance in persimmon that will be useful for breeding salinity tolerant rootstocks.

## Introduction

Persimmon (*Diospyros kaki* Thunb.) has become one of the most dynamic tree crops in the world. According to the data available (www.fao.org/faostat), global cultivated surface has increased 43% in the last 10 years (2006–2016) and world production increased 59%, which demonstrates an important improvement in crop yield. This trend has been highly relevant in some countries. For instance, in the Mediterranean basin, the cultivated surface has been increased by four times and production by near five [1]. Despite the recent and fast increase in persimmon production in the Mediterranean, the persimmon industry is facing important problems related to climate change. One of them is the soil salinization caused by the decrease and change of the rainfall distribution, which is causing an increase of salts in the irrigation water [2]. In order to keep the production in these areas, availability of rootstocks tolerant to salinity is required [3].

The most commonly used rootstocks for persimmon production in these areas are seedlings from *Diospyros lotus* species, because of its tolerance to lime-filled soils and its adaptability to the Mediterranean conditions. Furthermore, *D*. *lotus* has a root system that does not produce basal shoots [4], facilitating the management of the orchards. However, this species is highly sensitive to salinity [5,6]. Other species used as rootstocks in some countries is *Diospyros virginiana*. This species is tolerant to salinity and performs well on lime-filled soils, but confers too much vigor to the plant, and produces many basal shoots, thus hindering crop management [7,8]. The most used rootstock around the world are seedlings from *D*. *kaki*, which is not tolerant to salinity [9]. Additionally, *D*. *kaki* is highly sensitive to lime-filled soils and produces tap-roots with few lateral roots, which are rather fine and broke easily, all together makes difficult the plant management in the nurseries. Consequently, seeds from D. *kaki* are not commonly used in the Mediterranean Basin countries. On the other hand, *D*.*kaki* exhibits compatibility with all cultivars, whereas graft-compatibility in *D*. *virginiana* needs to be checked for each variety [4]. There is no data reported about Na^+^ toxicity in *D*. *kaki*, which can be accounted by an absence of high Na^+^ accumulation in the soils where they are cultivated or because the tolerance of tree plants to Na^+^. On the other hand, Cl^-^ accumulation has been reported problematic in persimmon for production and postharvest management [6,10]

In order to confer salinity tolerance rootstocks should be able to overcome the two components of salinity stress: the osmotic effects and ion-toxicity. Osmotic effects are caused by the total concentration of salt around the roots, which restricts water assimilation by roots and results in reduced plant growth. The osmotic stress immediately causes a response in the stomatal aperture of the plant mediated by abscisic acid, ABA [11]. On the other hand, ionic effects are caused by the accumulation of toxic concentrations of Na^+^ and Cl^-^ ions in plant tissues, causing premature organ senescence and tissue necrosis. To overcome these effects, plants use complex mechanisms including changes in morphology, water relations, photosynthesis, respiration and toxic ion distribution, among others [12]. Some studies related salinity stress with an increase of stored carbohydrate [13], causing a reduction in sink demand that may downregulate photosynthesis. Yet, it remains unclear if the reduction of growth rate causes a reduction of photosynthesis or vice versa [12]. The decrease of photosynthesis rate comes with an increase of reactive oxygen species (ROS) production. At reduced photosynthesis activity, photoinhibition might occur due to the light excess. Under this scenario, plants have two mechanisms to prevent oxidative damage of the photosystems: heat dissipation by pigments and electron transfer to oxygen acceptors. Genetic differences in salinity tolerance are probably not associated with differences in the ability of detoxifying ROS. Instead, they could be related to differences in stomatal closure or CO_2_ fixation, as these mechanisms are essential for plant survival under natural variable situations [12].

Other studies have reported the possible induction of K^+^ deficiency by Na^+^, together with Na^+^ and Cl^-^ causing tissue necrosis [3]. These effects are visible in older leaves [14,15], leaf margins [16], and epidermis [17–20] probably as result of an evolved mechanism for protecting photosynthetically active cells [21]. Neverthless, Na+ and Cl- accumulation lead to ion imbalances in the cytosol that cause several toxicities, even leading to the loss of photosynthetical pigments [22]. While the physiological effects of salinity are well characterized, the mechanism to explain how toxicity affects the cells remains unknown [12].

A reduction in root hydraulic conductance can be observed in roots grown with salt presence [23,24]. This effect might be related to aquaporin activity. They are membrane intrinsic proteins involved in transport of water and small neutral solutes through the cells [25]. According to its amino acid sequences and subcellular localizations, plant aquaporins are classified into four subfamilies: plasma membrane intrinsic proteins (PIPs), tonoplast intrinsic proteins (TIPs), NOD26-like intrinsic proteins (NIPs) and small basic intrinsic proteins (SIPs) [26]. In fact, it has been observed that reduction of the hydraulic conductance can be linked to a lowered plasma-membrane intrinsic protein (PIP) aquaporin activity [27]. Also, reduction in PIP aquaporin gene expression has been observed under salinity stress [27–29]. Interestingly, in citrus rootstocks, PIP expression has been reported to be higher in tolerant genotypes compared to sensitive ones [30]. However, experiments on yeast and *Xenopus oocytes* have shown a strong Na^+^ conductance of AtPIP2;1 from *Arabidopsis thaliana*, suggesting that orthologues of PIP2;1 may act as a gate for Na^+^ influx into the plant [31].

Prevention of the toxicity effect might be related to a mechanism of exclusion of toxic ions or their compartmentation. In this context, Na^+^ access to the plant vascular system is mediated by non-selective cation channels [32]. Once inside the outer part of the root, the majority of the Na^+^ is pumped out from the cells via plasma membrane Na^+^/H^+^ antiporters in a high energy demanding process [33]. In *Arabidopsis thaliana*, a plasma membrane encoding gene (*SOS1*) has been identified with Na^+^/H^+^ antiporter activity [34]. This gene has been also related to the elimination of Na^+^ from the xylem [35]. The *SOS1* gene is the final part of a proposed signal transduction pathway responsible of maintaining ion homeostasis during salt stress [36]. Under high concentrations of Na^+^ in the cytoplasm, Ca^2+^ increase is triggered. The excess of Ca^2+^ ions are bound with a myristoylated calcium-binding protein CBL4 (SOS3) that acts as a sensor to perceive the Na^+^ mediated Ca^2+^ spike. At this point, *CBL4* gene is able to interact with a serine/threonine protein kinase CIPK24 (SOS2) [37–40] that activates the target gene *SOS1* [41–45], activating the retrieval of Na^+^ from the cytosol. Furthermore, SOS pathway has been proposed to be part form a signaling network, and other genes might be implicated in activation of SOS pathway, such as *SCaBP8* or *MPK6*. Furthermore, *SOS2* and *SOS3* genes seem to induce changes in the cytoskeleton that would cause root architectural changes in order to overcome the saline stress [46]. The SOS pathway consumes plasma membrane H^+^ gradient, and increased SOS1 expression may increase Na^+^ tolerance, but at the expense of plant growth [47]. This mechanism of Na^+^ removal from apoplast to cytosol is particularly important in root tip cells, due to the lack of vacuoles [48].

Other genes have been related with Na^+^ exclusion from the xylem, such as some members of the *HKT* (High affinity potassium transporter) family [12] and *CHX* [cation/H^+^ exchanger] family [49]. In *Arabidopsis*, *AtHKT1* has been identified as a Na^+^ selective uniporter with some role in K^+^ transport [50]. Also, *hkt1;1 Arabidopsis* mutants showed hyper accumulation of Na^+^ at the shoots while showing less Na^+^ accumulation on the roots [51–53], suggesting a role on Na^+^ long transport via xylem and phloem [52,54]. Multiple isoforms have been isolated in monocots [55–58] and in several cereals HKTs can mediate Na^+^ uptake [47,59,60]. Under K^+^ starvation and Na^+^ stress, it has been observed increased transcript abundance of *AtCHX17* [61]. AtCHX23 and AtCHX20 have been located in the chloroplast envelope [62] and endosomal membranes [63], suggesting intracellular functions. However, CHX family genes might be limited to cellular K^+^ homeostasis [64], as experiments using *GsCHX19*.*3* from cotton have shown increased K^+^ deficiency tolerance in yeast [65]. NHX type antiporters have been also proposed to have a role in salt tolerance [66]. Its role seems to be related to maintaining Na^+^/K^+^ homeostasis rather than extruding or sequestrating Na^+^ from the cytosol. Furthermore, it seems to have also a crucial role in stomatal closure via turgor regulation at guard cells [67].

As the plants have complex Na^+^ exclusion pathways, Cl^-^ accumulation becomes potentially more toxic than Na^+^ accumulation. Cl^-^ influx into the plant has been proposed to depend on a passive mechanism via anion channels that are downregulated by ABA [12]. Chloride Channel (CLC) family has been found in the tonoplast of various plant species. Cation/Cl^-^ cotransporter (CCC) might be involved in Cl^-^ sequestration into other types of intracellular compartments [47].

Another strategy used by plants when the ion exclusion is not possible is the vacuole compartmentation of toxic ions. In *Arabidopsis*, Na^+^ compartmentation is believed to be carried out by Ca^2+^/cation exchangers (CCXs) as vacuolar Na^+^ sequestration [47]. In the case of Cl^-^, the role is taken by the ALMT (Aluminum-activated Malate Transporter) protein family that encodes anion transmembrane channels [68]. The *Arabidopsis* vacuolar H^+^-translocase pyrophosphatase (AVP) also has a role in pumping Na^+^ into the vacuole through enhancing the H^+^ electrochemical potential difference, improving salinity tolerance [69,70]. In *Arabidopsis*, tonoplast *ALMT9* gene knock-out mutants shown shoot accumulation of both Cl^−^ and Na^+^. On the other hand, *almt9* plants complemented with a mutant variant of *ALMT9* that exhibits enhanced channel activity showed higher Cl^−^ and Na^+^ accumulation [21], suggesting a role of *ALMT9* on ion compartmentation.

In this context, this study was aimed at identification of salinity tolerant rootstocks for persimmon production, combining the high salinity tolerance of *D*. *virginiana*, and the positive traits of *D*. *kaki*. For this purpose, a progeny (*D*. *virginiana* x *D*. *kaki*) x *D*. *kaki* was generated and phenotyped for salinity tolerance. The objectives are to explore the mechanisms involved in salinity tolerance in persimmon and develop alternative rootstocks for saline environments.

## Material and methods

### Plant material and salinity treatment

The *D*. *kaki* population (DK) was obtained from open pollination of female trees. The *D*. *virginiana* (DV) population was obtained from a single open pollinated tree. A third population was obtained from the cross between a *Diospyros kaki* genotype with male flowers used as a male parent and a hybrid tree obtained between D. kaki, as a male parent and *D*. *virginiana* as a female parent. Both progenitors of the hybrid tree were single individuals from open pollination. The population obtained is therefore (*D*. *virginiana* x *D*. *kaki*) x *D*. *kaki*) an interspecific backcross of D. kaki. At the end of March, seeds were stratified for 30 days in plastic bags filled with perlite in a cold chamber at 4°C. After stratification, seeds were transferred to trays containing peat-moss and perlite (4:1 ratio, respectively) and kept in a greenhouse at 18–24°C for two months (from April, 29, to June, 27, 2016). Sixty-five seedlings of each parental line and 420 seedlings of the BC line were transplanted into 1L pots containing coarse sand. The plants were randomly distributed in the greenhouse and watered with a nutrient solution (3% Cristaljisa 18-18-18, soluble fertilizer with micronutrients) during one week, to acclimate the plants before exposition to the salinity treatment. After the acclimation week the plants were submitted to a salinity treatment for 72 days (from July, 5, 2016 to September, 15, 2016). The treatment consisted in 40 mM NaCl added to the nutrient solution. The controls remained watered with the standard nutrient solution. The amount of NaCl added were already described in a previous experiment [9].

### Morphological phenotyping

All the plant material was phenotyped for the following variables: height (cm), leaves (no.), nodes (no.), internodes (cm) and defoliation (1-no. leaves/no. nodes). They were recorded at the beginning of the experiment (day 0) and at the end of the salinity treatment (day 72). The ratio between initial and final value of variables related to growth was also calculated. Based on visual symptoms, salinity injury was rated from 0 to 4: 0 –no symptoms, 1 –leaf turgor loss, 2 –leaf tip necrosis, 3 –leaf margin necrosis, 4 –defoliated plant. These data were used to divide the BC population into three groups according to its salt tolerance: tolerant, sensitive and intermediate phenotypes. Only tolerant and sensitive groups were used in further analyses.

### Leaf gas exchange parameters

Stomatal conductance (g_s_), leaf net CO_2_ assimilation rate (A_CO2_), leaf transpiration rate (E) and internal CO_2_ concentration (C_i_) were measured on single attached leaves from glasshouse-cultured plants. Intrinsic leaf water use efficiency (WUE) was calculated as A_CO2_ and g_s_ ratio. All measurements were carried out in a sunny day between 9:30 a.m. and 12:30 p.m. at the end of the salt treatment (day 72). Photosynthetically active radiation (PAR) at the leaf surface was adjusted to a photon flux density of 1.000 μmol m^-2^ s^-1^. A closed gas exchange CIRAS-2 (PP-systems, Hitchin, UK) was used for the measurements. Leaf laminae were fully enclosed within a PLC 6 (U) universal leaf autocuvette in a closed-circuit model and kept at 25 ± 0.5°C, with a leaf-to-air vapor deficit of about 1.7 kPa. The air flow rate through the cuvette was 0.5–1.5 L min^-1^. Determinations were performed using uniform fully expanded leaves from the mid-stem zone of each of 57 BC treated plants (28 tolerant and 29 sensitive), 15 of BC control, 19 DK treated, 9 DK control, 26 DV treated and 10 DV control.

### Leaf water relations

Leaf stem water potential ψ_H_, MPa) was measured in fully expanded leaves in a sunny day using a Model 600 Schölander Pressure Chamber (PMS Instrument Company, Albany, OR, USA) at the end of the salinity treatment (day 72), on the same plants used for the leaf gas exchange parameters. Previously, the leaf was kept in a reflective plastic bag for 30 minutes to remove water loss. For osmotic potential, after the same procedure, the leaf was introduced into microcentrifuge tubes and frozen immediately to -80°C for breaking the cells by ice crystallization. After 48h, frozen samples were centrifuged at room temperature to extract the cell sap (modified from Callister et al. [71]). Leaf osmotic potential (ψ_π_, MPa) of the leaf sap was calculated by van´t Hoff equation after measuring sap osmolarity (mmol kg^-1^) using an automatic osmometer (Wescor, Logan, USA). Leaf turgor potential (ψ_t_, MPa) was estimated as the difference between ψ_H_ and ψ_π_.

### Proline content and ion analysis

At the end of the treatment, adult leaves were collected from all survival plants from parental populations: treated and control DK (19 and 13, respectively), DV (26 and 15, respectively), and 32, 46 and 25 for tolerant, sensitive and control BC plants, respectively.

Proline content of leaves (mg g^-1^ of dry weight) was measured by the method of Bates et al. [72]. Dried leaves (250 mg) were homogenized in 1.5 mL of 3% (w/v) aqueous sulphosalicylic acid. The homogenate was centrifuged and 0.2 mL of supernatant was mixed with 0.7 mL of ninhydrin acid and 0.6 mL of glacial acetic acid. The mixture was incubated at 100°C for 1 h and the reaction was cooled in an iced bath. The chromophore was extracted using toluene and its absorbance at 520 nm was determined by spectrophotometry (Lambda 25, PerkinElmer, Shelton, CT, USA).

For ion analysis, collected samples were washed; fresh and dried (oven-dried for 48 h at 65°C) weight was recorded. Dried leaves were ground to powder. For chloride determination (mg Cl^-^ g^-1^ of dry weight), 25 mg of leaf powder was diluted in 20 mL of combined acid buffer (Sherwood Scientific Ltd. Cambridge. UK). Chloride concentration (mg mL^-1^) of the filtered solution was determined by silver ion-titration [73] with a Corning 926 automatic chloridometer (Corning Ltd. Halstead Essex, UK). A portion of dried leaves (0.5 g) were burnt in a muffle furnace for 12 h at 550°C. Remaining ashes were digested with HNO_3_ 1M solution. Na^+^, Ca^2+^, K^+^, Mg^2+^, P and S ions were quantified (mg g^-1^ dry wt) using a multiple-collector inductively coupled plasma mass spectrometry (MC-ICP MS, Thermo Finnigan Neptune).

### Gene expression analysis

A subset of each group was selected for gene expression analysis (Table 1). Root tip tissue was collected after 72 days of salt treatment and immediately frozen and powdered using liquid nitrogen. Control samples from the three populations were collected and processed. RNA was isolated according to Gambino et al. [74]. DNA was removed with the RNase-Free DNase Set (Qiagen, Valencia, CA, USA), using the RNeasy Plant Mini Kit (Qiagen). Purified RNA (500 ng) was reverse transcribed with PrimeScript RT Reagent Kit (Takara Bio, Otsu, Japan) in a total volume of 10 μL.

**Table 1:** Selected plants (tolerant and susceptible) for gene expression analysis.

	*D*. *virginiana*	*D*. *kaki*	Backcross line (BC)			
			BC_t_*		BC_s_**	
Treated plants	V10	K9	BC11	BC312	BC61	BC198
	V14	K23	BC61	BC315	BC77	BC236
	V20	K26	BC127	BC323	BC90	BC237
	V23	K34	BC175	BC359	BC95	BC301
	V37	K44	BC291	BC375	BC172	BC333
Untreated plants	V4	K4	BC2			
	V5	K6	BC5			
	V7	K7	BC16			
	V11	K9	BC22			
	V15	K14	BC25			

*BC_t_: tolerant backcross line

**BC_s_: susceptible backcross line

Eight putative orthologous genes involved in different mechanisms leading to salt tolerance were analyzed. *Arabidopsis* genes *SOS1* (AF_256224.1), *SOS2* (AF_237670.1), *SOS3* (HE_802983.1), *NHX1* (AF_106324.1), *HKT1* (AK_228564.1) and *ALMT9* (NM_112729.4) were blasted against the SRA archive of *D*. *lotus* (SRA ID: SRP045872) cv. Kunsenshi [75]. The output fragments were manually assembled to complete putative orthologous genes. Specific persimmon primers were designed using the sequences obtained (Table 2).

**Table 2:** Primers used for RT-qPCR analysis.

Gene name	Sequence (5’-3’)
SOS1-Like	F:GGATTTTCTCTGGAAGGAAAGTGCTA R:GGAGATGTAATCAGTTCCTCTTTGACAC
SOS2-Like	F:TTAGAGTTTGTTACTGGAGGGGAACT R:CACTCAGTCCAAAGTCAGAAACCTTCA
SOS3-Like	F:GAAGTTGAGGCCTTGTATGAGCTATTT R:CCTAATGAACGAACAAATTCTCCAAACTC
HKT1-Like	F:GATTCCTAACCCTGCAGATAAACCCATT R:GTTGCAGACACAGAGGTAAAGAACAAG
NHX1-Like	F:CACCAAAGAACTTGACAAGAATGCTG R:CCAATAGTAGTGCACGGTACGAG
ALMT9-Like	F:TCACTTATGCAAAACTATACCCCACAATG R:GTAGATAAACATATTCACCACCAAACACAC
PIP1 Family-Like	F:GTCTTCTACATGGTGATGCAGTGC R:AGTGGCAGAGAAGACAGTGTAGAC
PIP2 Family- Like	F:GCATGATCTTCATCCTCGTCTACTGCAC R:TTGGGATCAGTGGCGGAGAAGAC

For plasma membrane intrinsic (PIP) aquaporins, *Arabidopsis PIP1* (NM_001084854.2, NM_130159.4, NM_100044.5, NM_116268.4, NM_118469.4) and *PIP2* (NM_001035774.1, NM_129273.5, NM_129274.4, NM_125459.4, NM_115339.3, NM_129458.3, NM_001203991.1, NM_127238.3) family sequences were aligned and conserved regions within families identified. Each conserved region was blasted against *D*. *lotus* SRA archive. The output fragments were manually assembled and specific primers designed at the conserved region, obtaining specific primers for each putative aquaporin family (Table 2).

The first-strand cDNA was 60-fold diluted, using 1 μL as template in a final volume of 20 μL. Quantitative real-time PCR was performed on a StepOnePlus Real-Time PCR System (Life Technologies, Carlsbad, CA, USA), using SYBR premix Ex Taq (Tli RNaseH plus) (Takara Bio). The PCR protocol consisted of 10 min at 95°C, followed by 40 cycles of 15 s at 95°C, and 1 min at 60°C. The specificity of the reaction was assessed by the presence of a single peak in the dissociation curve and through size estimation of the amplified product by agarose electrophoresis. Four different genes were screened with Normfinder [76] for use as reference genes: *DkACT* [77], *DkUBC*, *DkPP2A*, and *DkTUA* [78] and two of them selected as reference: *DkACT* and *DkTUA*. The normalization factor was calculated by the geometric mean of the values of relative expression of both genes. Expression analysis was carried out in five treated and untreated DV and DK plants, 10 tolerant BC plants and 10 susceptible BC plants as biological replicates (Table 1). Results were the average of three technical replicates.

### Statistical analyses

Within treatment (saline vs saline and non-saline vs non-saline) parameters were statistically tested by Analysis of Variance (ANOVA) and averages were compared with the Least Significative Differences (LSD) method at 95% confidence level (P≤0.05). When comparing with the non-saline conditions, the parameters were found to not fit normal distribution and, therefore, were compared with Kruskal-Wallis test (P≤0.05) and median notch method [79]. Statgraphics Centurion, 16.1 version (Statistical Graphics, Englewood Cliffs, NJ, USA) was used for performing the statistical analyses. Principal component analysis (PCA) was carried out using S-Plus 8.0 (Insightful Corp., Seattle, USA). The variables included were: morphological traits, leaf gas exchange and leaf water relations parameters, proline and ion contents. The number of components retained was defined by the inflection point of the corresponding screen plot. A biplot of individual scores and loadings was obtained. An average plant for each population was included in the analysis representing the average of each variable for the population. Plants from the BC population were classified as tolerant or susceptible to salinity according to the phenotyping data and the distance to the average plant in the PC analysis.

## Results

### Populations phenotyping

**Fig 1:**
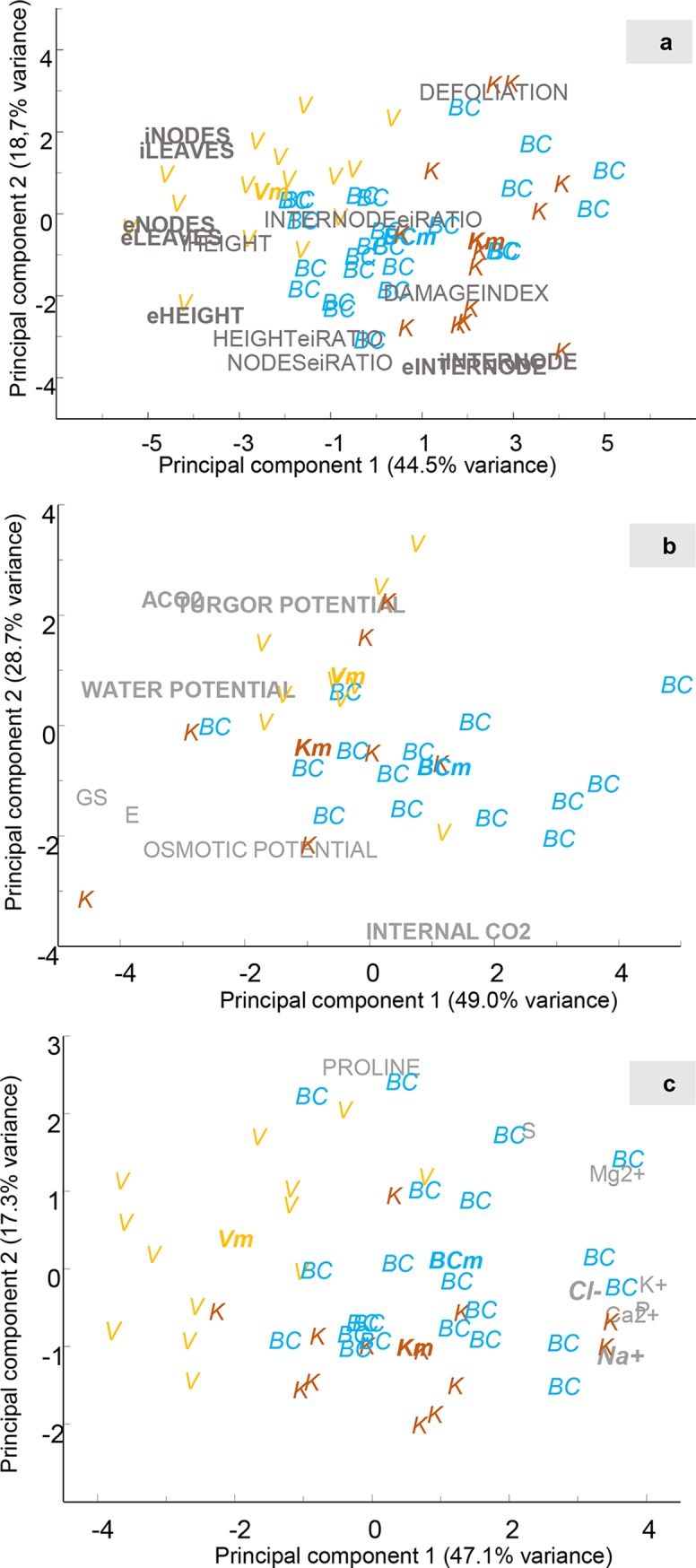
Plot of the first two components from a principal component analysis of morphological (a), leaf gas exchange and leaf water relations (b) and ionic and proline content (c) of the three populations in non-saline conditions. Each letter represents each population: V–*D*. *virginiana* population (yellow), K–*D*. *kaki* population (brown) and BC–Backcross population (blue). Vm, Km and BCm in bold represents the mean of the individuals. Gray letters represent each of the measured variables. The most important variables identified in each PCA are in bold type.

Control plants from the three populations studied: *D*. *virginiana* (DV), *D*. *kaki* (DK) and the backcross (BC) grown in non-saline conditions were measured to address differences among populations. The variables were studied using PCA in which 63.2% of the total variance was explained by the two first components (Fig 1A). The average value of each variable/population was included in the analysis (referred as average plant). Plants from DV were the tallest at both the initial (day 0) and at the end of the experiment (day 72). They also had more leaves and nodes, with shorter internode length than those from DK. Although differences in the speed of growth were not considerable (bold letters in the figure of variable loadings indicate significant differences, ANOVA p<0.05), plants from DV tended to show higher ending to initial height and nodes ratios. The plants from the BC resulted in values between DV and DK populations. In fact, the mean average plant of BC was closer to DK than to DV population. This distribution was expected attending to the higher participation of the *D*. *kaki* genome in the backcross.

The differences in leaf gas exchange and leaf water relations followed similar pattern. (Fig 1B) with DV plants exhibiting higher A_CO2_ and lower C_i_ than DK and BC plants. No significant differences (non-bold letters) were observed in g_s_ and E and, ψ_π_ between the three populations and BC plants had lower ψ_H_ and ψ_t_. The accumulation of salt, nutrients (Cl^-^, Na^+^, Ca^2+^, K^+^, Mg^2+^, P, S) and proline in the leaves showed a similar pattern to that observed for morphological data. Plants from DV population were the most different in the PCA plot, while BC and *D*. *kaki* population plants were grouped (Fig 1C). For these variables PC1 and PC2 explained 46.7% and 17.3% of variability, respectively. DV plants accumulated lower amounts of ions (especially of Cl^-^, Na^+^, Ca^2+^, P and K^+^) and higher amounts of proline than DK and BC plants.

### Evaluation of tolerance to salinity

**Fig 2:**
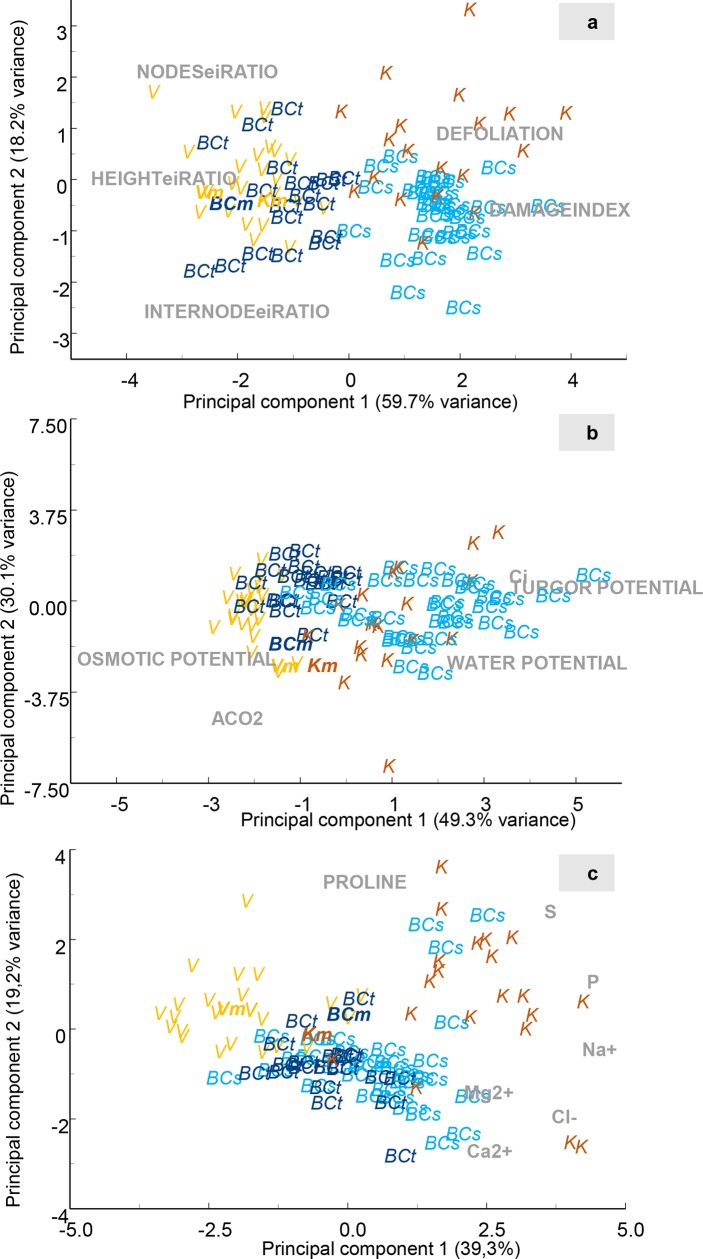
Plot of the first two components from a principal component analysis of morphological variables (a), leaf gas exchange and leaf water relations (b) ionic and proline content (c) of the three populations under saline conditions. Each population represented by letters: V–*D*. *virginiana* population (yellow), K–*D*. *kaki* population (brown) and BC–Backcross population (blue). BCt are the Backcross plants that showed a salt tolerant phenotype and BCs the plants salt sensitive. Vm, Km and BCm in bold represents the mean of the individuals under control treatment. Gray letters represent each of the measured variables.

A subset of 127 plants from each population (DV, *DK* and BC) were grown under saline conditions (40mM) to evaluate tolerance to salinity. The variables studied were height, nodes number, internode length, defoliation and damage index. All measurement aimed at addressing the effect of salinity on growth rates and plant damages (Fig 2A). Values of ending/initial (e/i) ratio were used. The height, number of nodes and internode length were selected as variables for rating the saline stress effect on plant growth. The initial and end values were excluded in the PCA, as the differences between the populations in non-saline conditions were considerable and would mask the specific effect of salinity (Fig 1A). In the PCA, mean values of the average plant corresponding to non-saline conditions were included to enable a comparison between non-saline and saline conditions.

**Fig 3:**
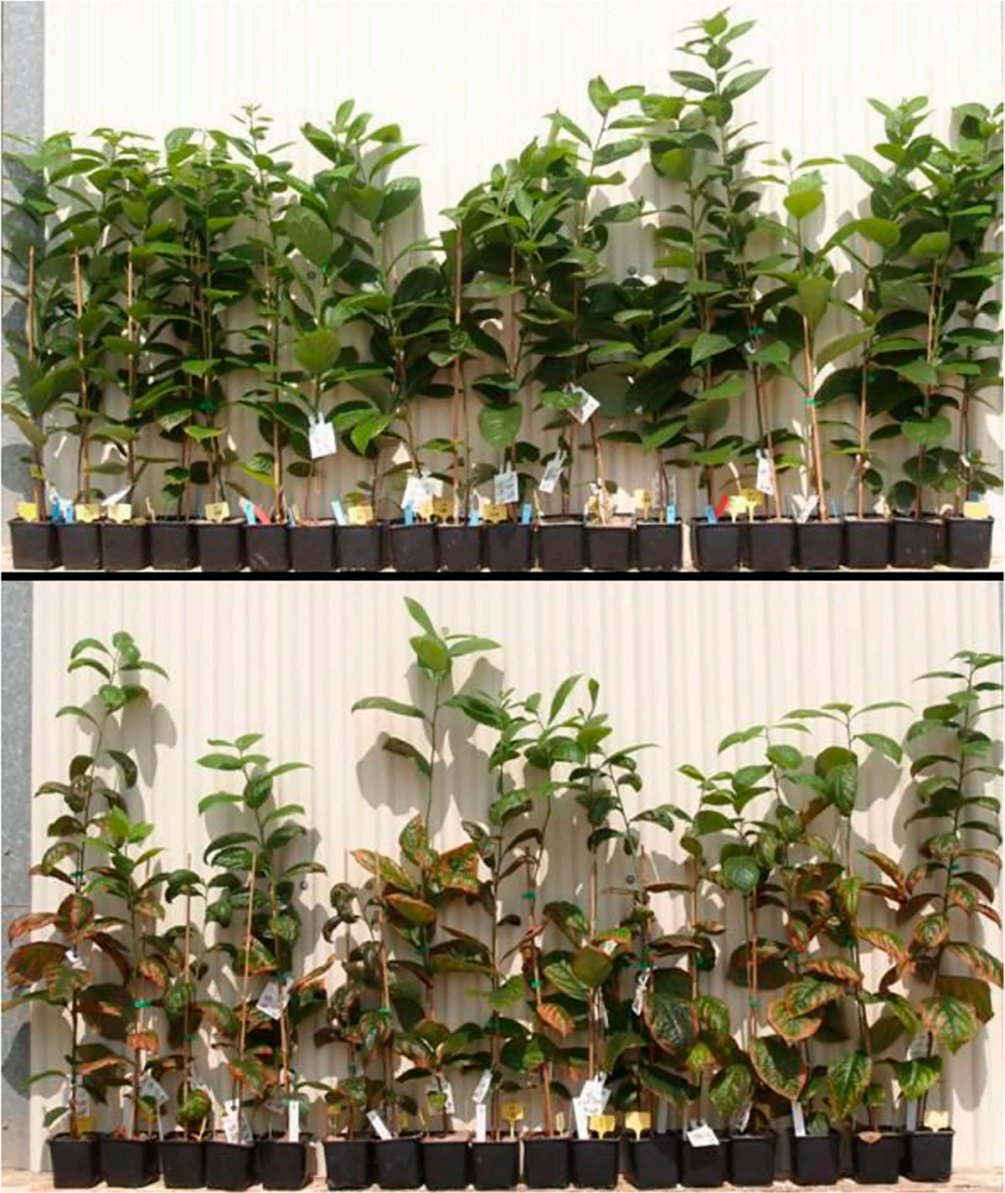
Phenotype of the saline tolerant (up) and sensitive (down) backcross population (BC) plants after 72 days of irrigation with 40mM NaCl.

The tolerance to salinity of DV population plants was evident. In the analysis of PCA related to morphological variables the first two components explained 77.9% of variance (Fig 2A). DV plants under saline conditions were morphologically similar to those under non-saline conditions (V_m_), reflecting that the growth rate and damages were not considerably altered by saline treatments. On the other hand, DK plants showed high susceptibility to salinity, with lower growth rates and higher levels of defoliation and damage under saline conditions compared to non-saline ones (K_m_) (Fig 2A). BC plants treated with salinity differed significantly from those under non-saline conditions. BC treated with salinity plants distribution overlapped with DV and DK plants distribution. According to PC1 some plants showed similar behaviour than DV under saline conditions and some were located close to the BC average plant under non-saline conditions (BC_m_), resembling the behaviour of the susceptible DK population plants (Fig 2A). Based on these results, BC plants were classified as tolerant (BC_t_) and susceptible (BC_s_) to salinity (Fig 3). The tolerance of DV, the susceptibility of DK, and the tolerance and susceptibility of BC plants were confirmed with ANOVA tests (Table 3). The values and the ratios between them and the average values in non-saline conditions (saline/non-saline ratios, s/ns ratios) were used. DV plants showed values for morphological variables similar to those obtained in control conditions, being the s/ns ratios close to 1 for most variables (Table 3). The s/ns ratio for damage index (1.79 folds) revealed more damage under salinity, although the overall damage by salinity was low (0.13) (Table 3).

**Table 3:** Phenotype of each population under saline and non-saline conditions for all the measured variables. Morphological variables are expressed as the ratio of each individual at the end of the treatment (saline conditions) and the beginning of the experiment (non-saline conditions). Different letters represent significant differences between populations (p<0.05). n(treated/control) indicated the number of plants measured in every experiment.

	*D*. *kaki*	*D*. *kaki x D*. *virginiana*		*D*. *virginiana*
		Sensible	Tolerant	
Agro-morphological data	Mean (s/ns ratio*)	Mean (s/ns ratio)	Mean (s/ns ratio)	Mean (s/ns ratio)
Initial Height (iH, cm)	17.22^a^ (1.15^ab^)	33.35^c^ (2.23^c^)	23.69^b^ (1.38^b^)	21.00^ab^ (1.06^a^)
End Height (eH, cm)	42.21^a^ (0.62^a^)	82.51^b^ (1.22^d^)	92.16^bc^ (1.05^c^)	92.63^c^ (0.87^b^)
Height_ei_Ratio (eH:iH)	2.44^a^ (0.55^a^)	2.48^a^ (0.56^a^)	4.01^b^ (0.78^b^)	4.46^c^ (0.83^b^)
Initial Leaves (iL, n°)	5.58^a^ (1.00^a^)	10.85^c^ (1.96^c^)	8.84^b^ (1.32^b^)	10.54^c^ (1.01^a^)
End Leaves (eL, n°)	13.63^a^ (0.77^a^)	24.79^b^ (1.41^c^)	28.37^b^ (1.10^b^)	38.54^c^ (0.96^ab^)
Initial Nodes (iN, n°)	5.57^a^ (1.01^a^)	11.00^c^ (1.99^b^)	9.00^b^ (1.28^c^)	10.54^c^ (1.01^a^)
End Nodes (eN, n°)	16.11^a^ (0.84^a^)	27.55^b^ (1.44^c^)	30.11^b^ (1.10^b^)	40.50^c^ (0.94^ab^)
Nodes_ei_Ratio (eN:iN)	3.04^b^ (0.84^b^)	2.49^a^ (0.69^a^)	3.41^c^ (0.87^bc^)	3.88^d^ (0.94^c^)
Initial Internodes (iI, cm)	3.27^c^ (1.14^a^)	3.04^c^ (1.06^a^)	2.71^b^ (1.10^a^)	2.02^a^ (1.04^a^)
End Internodes (eI, cm)	2.59^a^ (0.73^a^)	3.02^b^ (0.85^b^)	3.16^b^ (0.99^c^)	2.32^a^ (0.92^bc^)
Internode_ei_Ratio (eI:iI)	0.82^a^ (0.65^a^)	1.00^b^ (0.80^b^)	1.19^c^ (0.89^c^)	1.16^c^ (0.86^bc^)
Defoliation (eN:eL)	0.17^c^ (2.07^c^)	0.11^b^ (1.35^b^)	0.06^a^ (0.96^ab^)	0.05^a^ (0.71^a^)
Damage Index	2.26^b^ (4.19^b^)	2.67^c^ (4.94^b^)	0.05^a^ (0.26^a^)	0.13^a^ (1.79^a^)
n (treated/control)	28/13	53/15	38/15	42/15
**Leaf gas exchange**				
A_CO2_ (μmol CO_2_ m^-2^ s^-1^)	3.01^a^ (0.44^a^)	2.96^a^ (0.45^a^)	4.23^a^ (0.64^ab^)	6.67^b^ (0.74^b^)
g_s_ (mmol H_2_O m^-2^ s^-1^)	51.74^c^ (0.86^bc^)	47.79^bc^ (0.96^c^)	29.58^a^ (0.59^a^)	41.75^b^ (0.74^ab^)
E (mmol H_2_O m^-2^ s^-1^)	1.37^b^ (0.86^b^)	1.28^b^ (0.91^b^)	0.77^a^ (0.55^a^)	0.82^a^ (0.54^a^)
C_i_ (μmol CO_2_ m^-2^ s^-1^)	304.90^b^ (1.67^b^)	278.94^b^ (1.67^b^)	163.58^a^ (0.98^a^)	124.17^a^ (1.09^a^)
n (treated/control)	19/9	29/15	28/15	26/10
**Leaf water relations**				
Water pot. (ψ_H_, MPa)	-0.71^b^ (1.33^b^)	-0.68^b^ (0.78^a^)	-1.04^a^ (1.20^b^)	-1.13^a^ (1.63^c^)
Osmotic pot. (ψ_π_, MPa)	-2.53^b^ (1.59^b^)	-3.09^a^ (1.91^c^)	-2.40^b^ (1.48^b^)	-2.06^c^ (1.23^a^)
Turgor pot. (ψ_t_, MPa)	1.83^c^ (1.72^b^)	2.42^d^ (3.21^c^)	1.36^b^ (1.81^b^)	0.93^a^ (0.94^a^)
n (treated/control)	19/9	29/15	28/15	26/10
**Proline** (mg g^-1^ dry wt)	2.14^b^ (1.35^b^)	1.41^a^ (0.67^a^)	1.44^a^ (0.69^a^)	2.56^b^ (1.12^b^)
n (treated/control)	19/13	46/25	46/25	26/15
**Ion analysis**				
Cl^-^ (mg L^-1^)	2.50^c^ (11.05^c^)	2.59^c^ (9.43^b^)	2.00^b^ (7.28^a^)	1.18^a^ (6.38^a^)
Na^+^ (mg g^-1^ dry wt)	1.85^d^ (13.43^b^)	1.41^c^ (13.05^b^)	0.44^b^ (4.10^a^)	0.23^a^ (4.13^a^)
Ca^2+^ (mg g^-1^ dry wt)	0.39^ab^ (0.73^a^)	0.43^b^ (0.83^ab^)	0.50^c^ (0.97^c^)	0.34^a^ (0.88^bc^)
K^+^ (mg g^-1^ dry wt)	2.60^c^ (1.07^b^)	2.12^b^ (0.83^a^)	2.56^c^ (1.00^b^)	1.81^a^ (1.09^b^)
Mg^2+^ (mg g^-1^ dry wt)	0.11^b^ (0.98^c^)	0.09^a^ (0.63^a^)	0.08^a^ (0.57^a^)	0.09^a^ (0.81^b^)
P (mg g^-1^ dry wt)	1.40^d^ (1.70^c^)	0.75^c^ (0.78^a^)	0.62^b^ (0.64^a^)	0.38^a^ (0.97^b^)
S (mg g^-1^ dry wt)	0.17^c^ (1.43^c^)	0.11^b^ (0.85^b^)	0.08^a^ (0.66^a^)	0.09^ab^ (0.86^b^)
n (treated/control)	19/13	46/25	32/25	26/15

* s/ns ratio (saline/non-saline ratio): ratio between value at the end of the treatment and the average value in non-saline conditions *D*. *kaki* population plants were very affected by salinity with high values of defoliation (0.17) and damage index (2.26) (Table 3).

The classification of the BC plants according to the PCA was validated with ANOVA analysis. The BC group classified as tolerant (BC_t_) showed values of the s/ns ratios of e/i ratios statistically equal to those of the DV population, while the performance of the BC susceptible group (BC_s_) was closer to the DK population (Table 3). BC_s_ and BC_t_ plants exhibited a decrease in growth speed compared to the control plants, with s/ns ratios lower than 1 for height, nodes and internodes (Table 3), being those values corresponding to BC_t_ significantly higher than those of BC_s_. Additionally, BC_s_ plants exhibited a significant increase in defoliation and damage index, with values of 0.11 and 2.67, respectively, compared to BC_t,_ with values of 0.06 and 0.05, respectively (Table 3).

Regarding leaf gas exchange and leaf water relations parameters, the variability explained by the two first components was 79.4%. The plot showed different distribution between the populations of DV and DK (Fig 2B). This difference was not so evident under non-saline conditions (Fig 1B), thus reflecting that DV population exhibited a clear response to salinity. BC_t_ plants plotted within DV plants, while most BC_s_ plants plotted within the DK ones.

**Fig 4:**
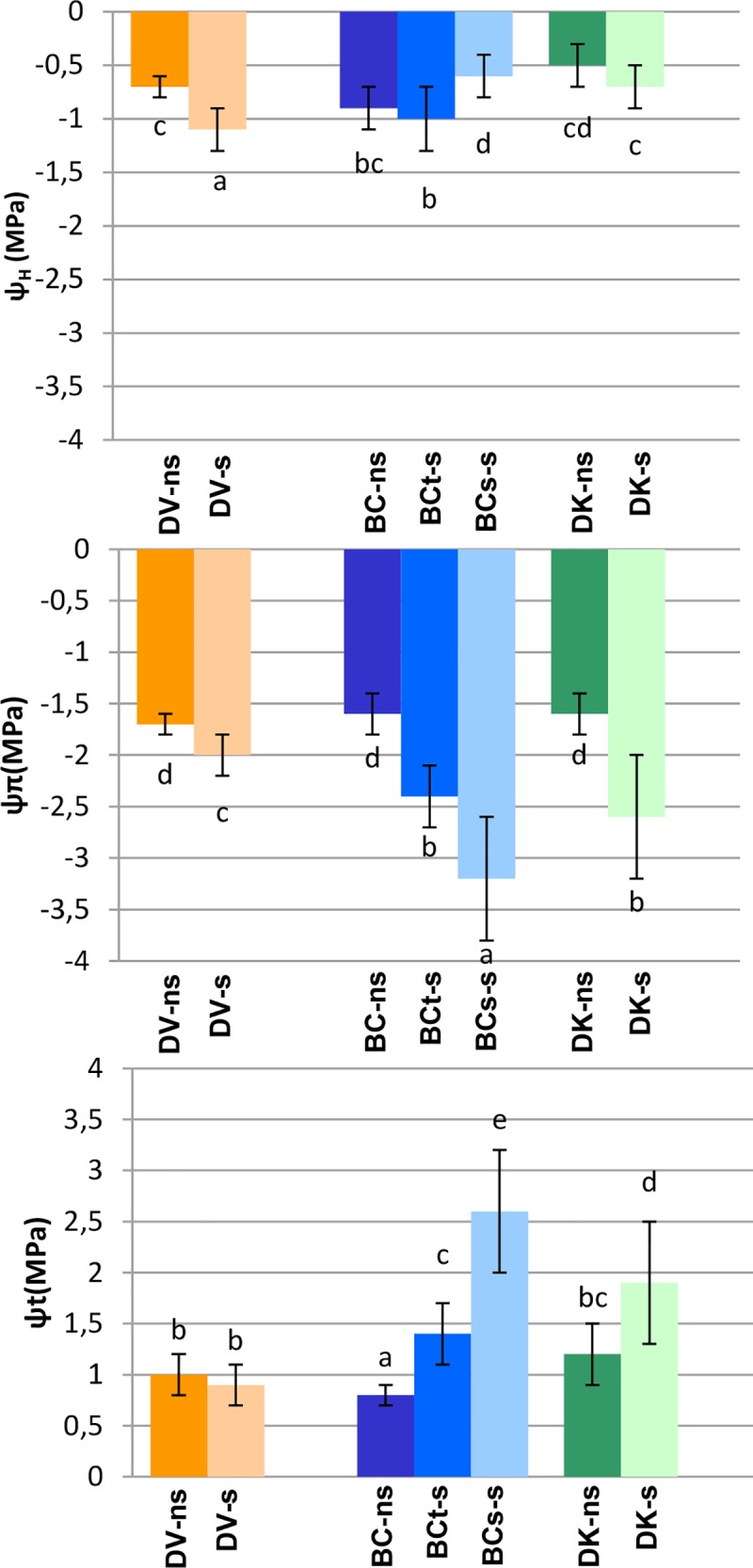
Leaf water relations measured on a pool of samples from each population under saline (s) and non-saline (ns) conditions: V–*D*. *virginiana* population (orange), K–*D*. *kaki* population (green) and BC–Backcross population (blue). The number of plants measured were 57 BC treated plants (28 tolerant and 29 sensitive), 15 of BC control, 19 DK treated, 9 DK control, 26 DV treated and 10 DV control. The vertical bars represent standard deviation. Different letters represent significant differences (p>0.05).

Almost all s/ns ratios of leaf water relations parameters (ψ_H_, ψ_π,_ ψ_t_) were higher than 1 in all the plants (Table 3). Only in the case of DV the ψ_t_ ratio was similar to non-saline conditions and in the case of BC_s_ plants the ψ_H_ ratio was lower than 1. DV plants exhibited significantly lower values of ψ_H_ and ψ_t_, and significantly higher values of ψ_π_ than the rest of plants, while BC_s_ plants showed significantly lower ψ_π_ and significantly higher ψ_H_ and ψ_t_ (Fig 4, Table 3).

**Fig 5:**
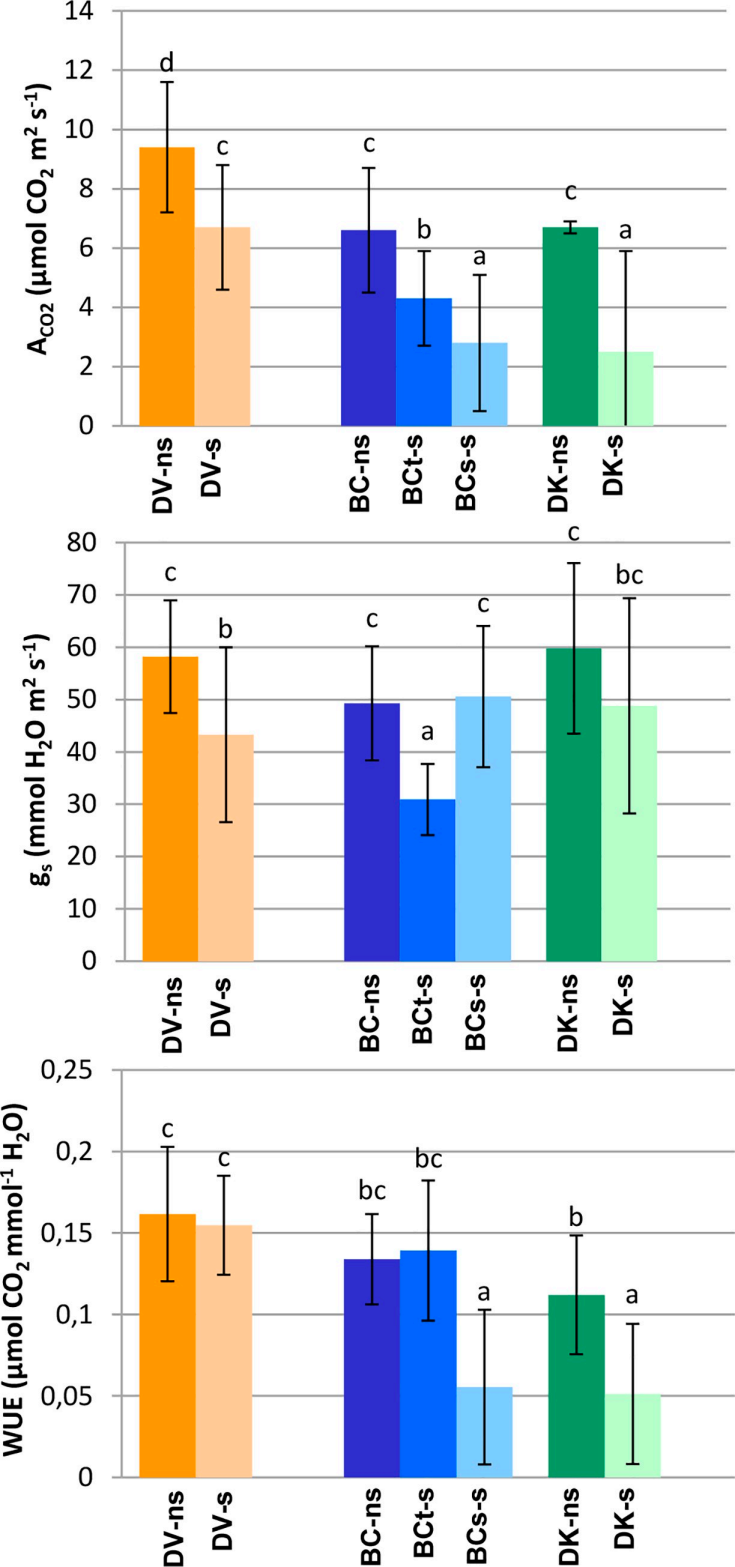
Leaf net CO_2_ assimilation rate (A_CO2_), stomatal conductance (g_s_) and intrinsic leaf water use efficiency (WUE), measured on a pool of samples from each population under saline (s) and non-saline (ns) conditions: V–*D*. *virginiana* population (orange), K–*D*. *kaki* population (green) and BC–Backcross population (blue The number of plants measured were 57 BC treated plants (28 tolerant and 29 sensitive), 15 of BC control, 19 DK treated, 9 DK control, 26 DV treated and 10 DV control. The vertical bars represent standard deviation. Different letters represent significant differences (p>0.05).

The population of DV and BC_t_ subset under salinity conditions showed a reduction of the A_CO2_, g_s_ and E (s/ns ratios lower than 1), while the C_i_ had a similar value to control conditions (Fig 5, Table 3). The reduction of s/ns ratio experimented by DK population and BC_s_ plants was significantly higher in the case of A_CO2_ but lower in the case of g_s_ and E, which showed values similar to the control conditions (Table 3). On the other hand, in these plants the C_i_ was higher under saline conditions (s/ns ratio > 1). DV plants had higher values of A_CO2_ and g_s_, compared to BC_t_ (Fig 5). No significant differences were found between DK and BC_s_ for leaf gas exchange parameters (Table 3). Under salinity conditions the leaf WUE of DV and BC_t_ plants was similar to the non-saline plants; however, it was decreased in *D*. *kaki* population BC_s_ plants (Fig 5).

Regarding leaf salt and nutrients (Cl^-^, Na^+^, Ca^2+^, K^+^, Mg^2+^, P, S) and proline accumulation, the first two components of the PCA explained 58.8% of total variance. DV plants was separated from the DK ones. DV plants plotted near its average plant under non-saline conditions (Vm), suggesting that these variables were not greatly affected by saline conditions. On the other hand, DK plants plotted away from its average plant under non-saline conditions (Km). The plants from the BC spanned between both populations without a clear differentiation between BC_s_ and BC_t_ plants (Fig 2C).

**Fig 6:**
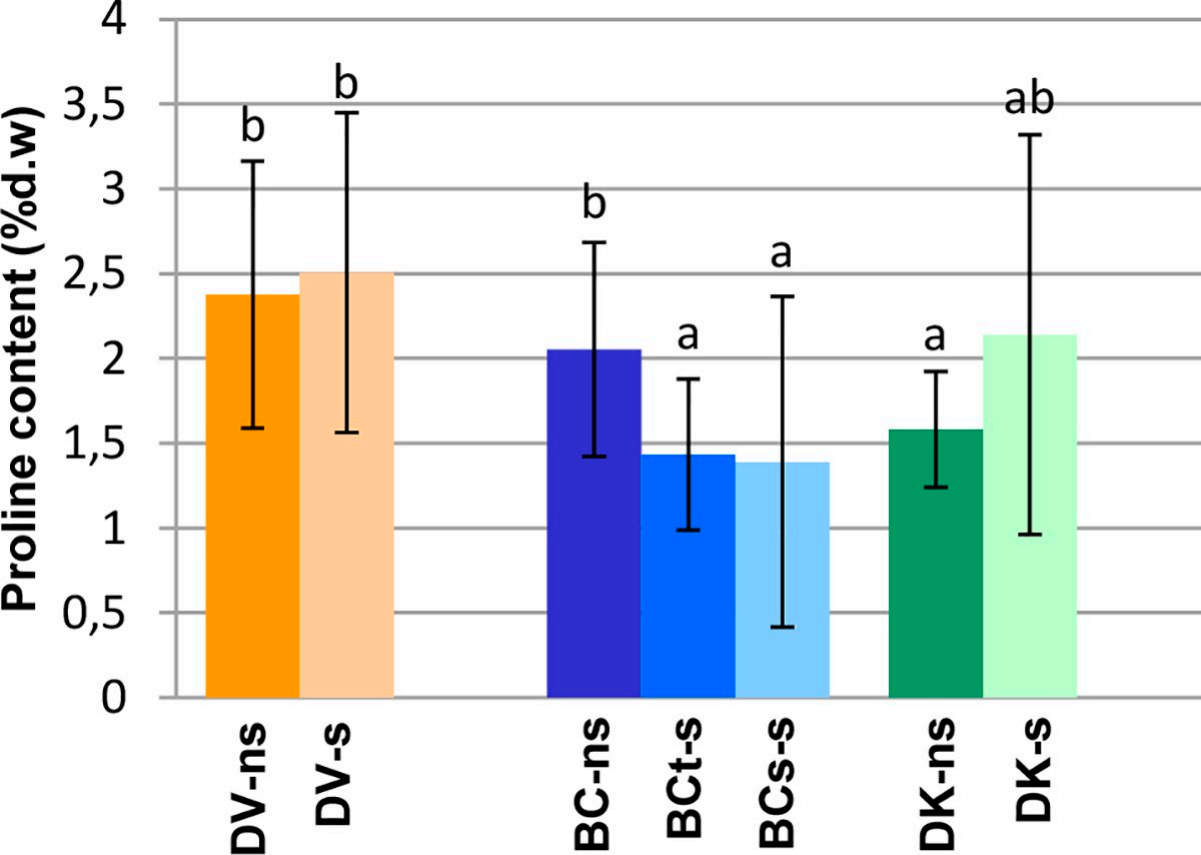
Leaf proline content on a pool of samples from each population under saline (s) and non-saline (ns) conditions: V–*D*. *virginiana* population (orange), K–*D*. *kaki* population (green) and BC–Backcross population (blue). The number of plants measured were treated and control DK: 19 and 13, respectively; DV: 26 and 15, respectively; 32 tolerant BC, 46 sensitive BC plants and 25 BC contro. The vertical bars represent standard deviation. Different letters represent significant differences (p>0.05).

In the case of proline, DK plants tended to accumulate higher amounts under saline conditions (1.35 fold), while BC_s_ and BC_t_ plants accumulated lower amounts (0.67 and 0.69 fold respectively) and DV plants tended to accumulate similar amounts (1.12 fold) in saline and non-saline conditions (Table 3). Plants from DK and DV populations showed significantly higher proline content than BC_s_ and BC_t_ plants (Fig 6).

**Fig 7:**
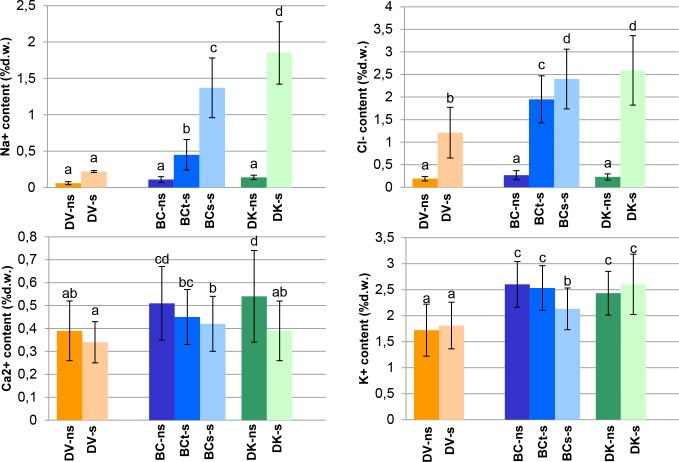
Na^+^, Cl^-^, K^+^ and Ca^2+^ leaf content on a pool of samples from each population under saline (s) and non-saline (ns) conditions: V–*D*. *virginiana* population (orange), K–*D*. *kaki* population (green) and BC–Backcross population (blue). The number of plants measured were treated and control DK: 19 and 13, respectively; DV: 26 and 15, respectively; 32 tolerant BC, 46 sensitive BC plants and 25 BC control. The vertical bars represent standard deviation. Different letters represent significant differences (p>0.05).

All populations showed s/ns ratios of Cl^-^ and Na^+^ contents higher than 1. Concerning to Ca^2+^ and K^+^ contents the s/ns ratios were close to 1, thus they were not affected by saline conditions (Table 3). DK and BC_s_ plants exhibited significantly higher values of Cl^-^ and Na^+^ when compared to DV and BC_t_ plants (Fig 7). The highest mean content of Ca^2+^ was found in the leaves of BC_s_ and BC_t_, while the highest contents of K^+^ and Mg^2+^ were found in the leaves of DK (Table 3, Fig 7).

### Gene expression analysis

**Fig 8:**
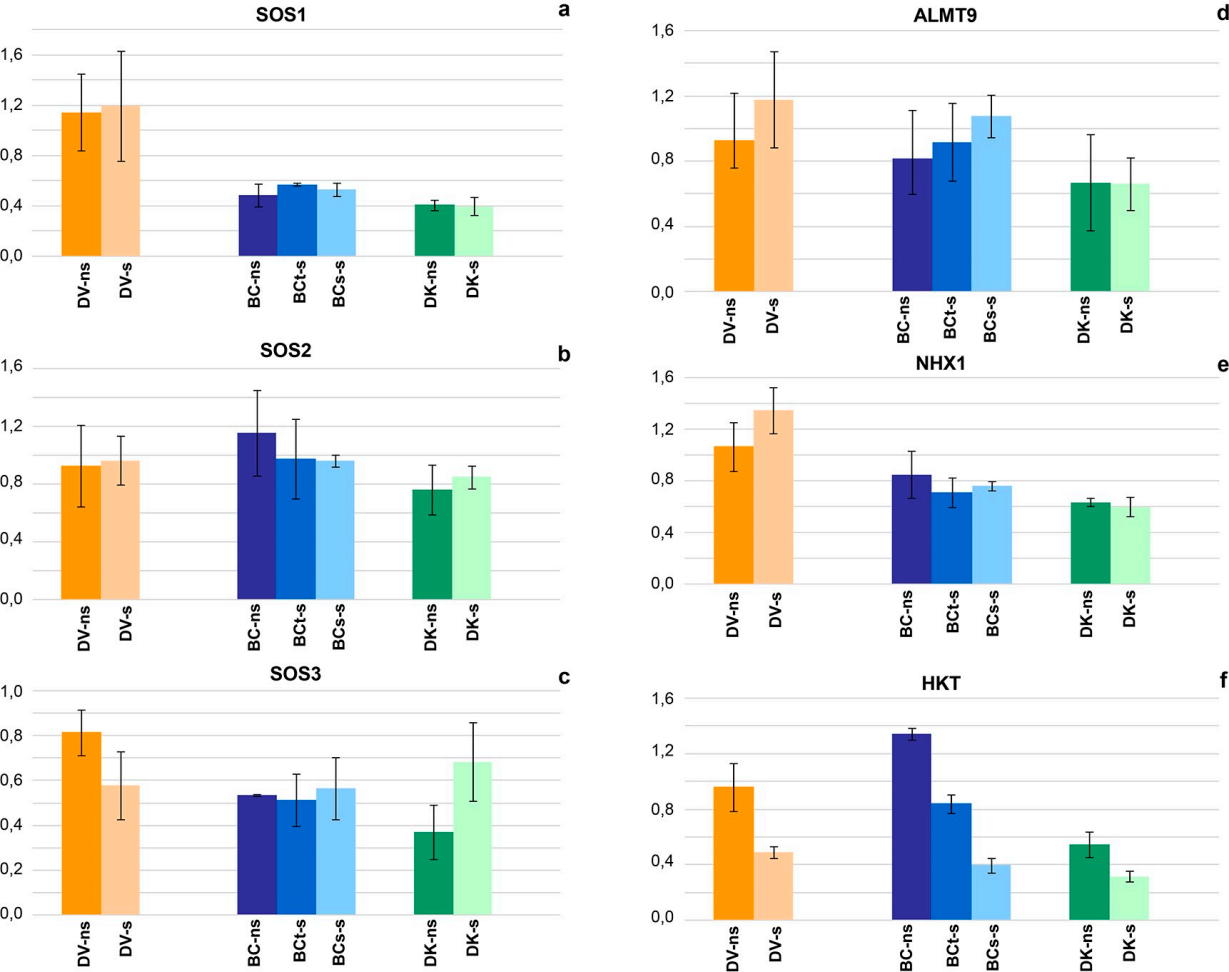
Relative expression of the genes SOS1-like (a), SOS2-like (b) and SOS3-like (c) measured on a pool of samples of each population under saline (s) and non-saline (ns) conditions: V–*D*. *virginiana* population (red), K–*D*. *kaki* population (green) and BC–Backcross population (blue). The vertical bars represent standard deviation.

In the case of the salt overly sensitive pathway SOS the differences in the expression level of *SOS2* and *SOS3* between populations were limited, being the expression of *SOS1* higher under both saline and non-saline conditions for the DV plants compare to DK and BC (Fig 8A, 8B and 8C). Regarding the comparison of gene expression between saline and non-saline conditions, no differences were found in the expression levels of *SOS2* and *SOS1* for DV, DK and the BC plants (Fig 8A and 8B). In the case of *SOS3*, DK showed expression levels considerably higher under saline conditions, while DV showed slightly reduced expression under saline conditions and no differences were found in the BC groups (Fig 8C).

In the case of the anion vacuolar channel *ALMT9*, the expression levels were lower in DK both in saline and no saline conditions compared to DV and BC, but the effect of saline conditions was not significant for any of them (Fig 8D). The expression levels of the Na^+^/H^+^ antiporter *NHX1* in DV was again higher than DK and the BC populations (Fig 8E). In the last two cases salinity did not increase the expression level, whereas in the case of DV the expression increased under saline conditions (Fig 8E).

A different pattern was found in the high affinity potassium transporter *HKT*, where the expression levels under non-saline conditions was higher for BC as compared to the rest of the populations under saline or non-saline conditions (Fig 8F). Saline conditions showed a reduction of expression levels of *HKT* from 40% to 50% in all cases. Interestingly, BC tolerant and susceptible plants showed a significant reduction in the expression levels under saline conditions, although the level of reduction was significantly higher for the susceptible ones. The expression levels of DV and BC_t_ plants under salinity were higher than those of DK and BC_s_ plants under similar treatment (Fig 8F).

**Fig 9:**
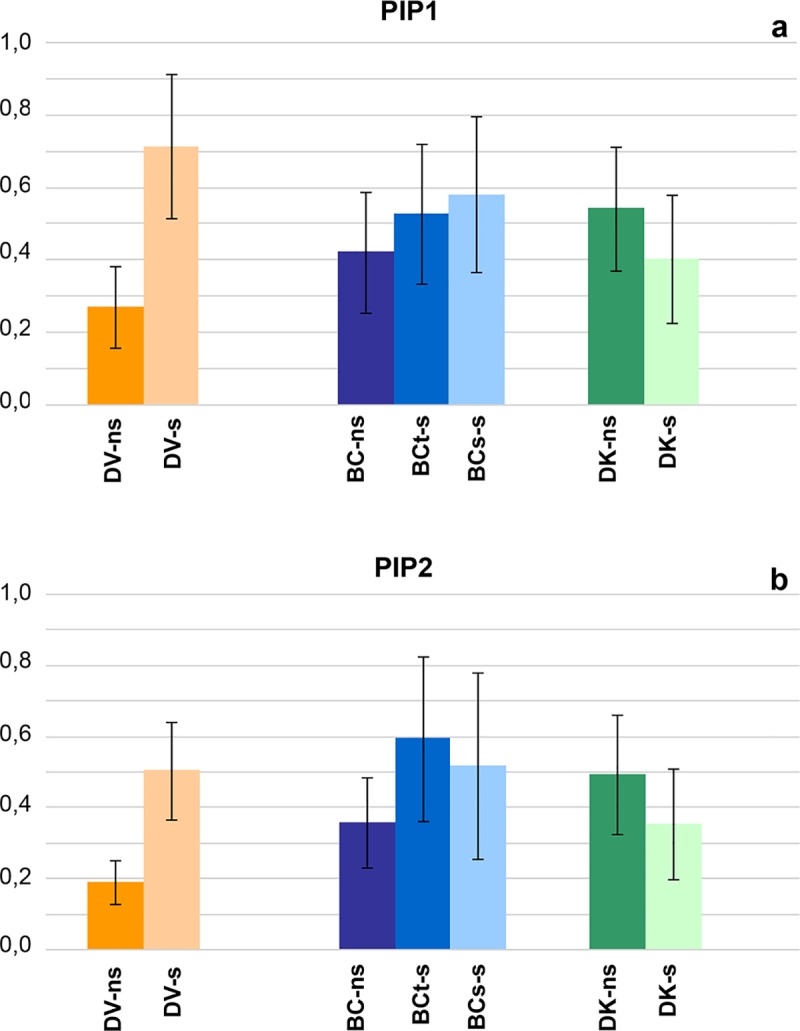
Relative expression of the PIP1-like (a), PIP2-like (b) families measured on a pool of samples of each population under saline (s) and non-saline (ns) conditions: V–*D*. *virginiana* population (orange), K–*D*. *kaki* population (green) and BC–Backcross population (blue). The vertical bars represent standard deviation.

In reference of plasma membrane intrinsic proteins PIPs, the expression levels of both *PIP1* and *PIP2* genes in DV were higher under saline conditions, while in DK the expression were slightly reduced by salinity (Fig 9). No differences were found in the expression levels of *PIP1* and *PIP2* in the BC tolerant and susceptible plants under saline conditions, while the expression under non-saline conditions was reduced (Fig 9).

## Discussion

Previous studies have analyzed the mechanisms behind salt tolerance in different species. In those studies multiple morphological, physiological and biochemical changes are described as responsible of plant adaptation to salinity [22]. Experiments of salinity tolerance using saline water have shown differential responses between species of the same genus, and between cultivars of the same species [14,80].

The *Diospyros* genus includes more than 400 species while only three species are widely used as rootstock: *D*. *lotus*, *D*. *virginiana* and *D*. *kaki* [4]. Species of the genus *Diospyros* show different degrees of tolerance to salt stress. *D*. *lotus* is the most common rootstock used for persimmon propagation in the Mediterranean basin. However, important damages attributed to ion toxicity has been reported in persimmon orchards grafted on *D*. *lotus*, which points out the need of selection of salinity tolerant rootstocks adapted to the Mediterranean environments [6,81]. *D*. *kaki* is the most used in persimmon orchards around the world because the affinity with all cultivars [4] and the lack of salinity presence in the areas where persimmon is mostly grown. On the other hand, *D*. *virginiana* has been described as more salt tolerant than *D*. *kaki* [7]. In a context of climate change and increase of salinity in soil and irrigation water, selections of tolerant rootstocks are required for maintaining the crop yield, or even improve it. In this study, salinity tolerance has been evaluated in plants from *D*. *virginiana*, *D*. *kaki* and a backcross population (BC) between both species aiming at identifying rootstocks tolerant to salinity to cultivate persimmon. Analyses of the effects on morphology, physiological parameters and gene expression after saline treatment were conducted to elucidate the mechanisms of tolerance to salinity.

### Effects on morphology

The effects of salinity on growth rate have been widely reported in different species [14]. The main effect of stress on plants is the progressive inhibition of growth as a consequence of an osmotic effect, that reduces the ability of the plant to absorb water, and a toxic effect by salt accumulation, that can produce the necrosis of leaves reducing the total photosynthetic leaf area [82]. At the end of the treatment with saline water, our results showed inhibition of vegetative growth in the populations studied; indicated by a decrease at three morphological variables: plant height, number of leaves and nodes and internodes length. Moreover, the responses differed significantly between populations. *D*. *virginiana* (DK) population was less affected than *D*. *kaki* (DK). After the salt treatment, some BC plants showed severe symptoms on plant growth and were classified as susceptible (BC_s_), while others showed moderate symptoms and were classified as tolerant (BC_t_). This fact indicates the presence of diversity within the BC plants related to the response to salinity that enables breeding for salinity tolerance.

### Osmotic stress responses

In salinity conditions, growth rate reduction could be a consequence of an inadequate photosynthetic activity, as a result of stomatal and non-stomatal factors [14]. All populations studied showed a reduction in the A_CO2_ compared to controls. In treated plants, salinity induced stomatal closure and reduction of C_i_ (Table 3 and Fig 5), similarly to the effects described in other species [83]. In persimmon, besides of differences between saline treated and control plants, persimmon tolerant genotypes showed a significant higher reduction of g_s_ and E compared to sensitive genotypes. Interestingly, under salinity conditions, the tolerant populations maintained values of WUE similar to the control, which means that the reduction of A_CO2_ and g_s_ was proportional (Fig 5), indicating that stomatal closure in tolerant genotypes is the main limiting factor of photosynthesis. These responses are mechanisms described for adaptability to osmotic stress caused by excessive salt environments [84]. Reduction of g_s_ could be used as an indicator of the tolerance to osmotic stress in these species [85]. Changes in g_s_ are always accompanied by changes in leaf water relations [86,87]. In agreement with previous studies in other species, significant differences in ψ_H_ between tolerant and sensitive populations were found in persimmon. The higher values of ψ_H_ found in tolerant plants (DV and BC_t_) indicates that salinity conditions affect much more the plant water status in tolerant than in sensitive plants. This differential response was attributed to the mechanism of osmotic adjustment developed in sensitive plants, favored by the higher accumulation of ions such as Cl^-^, that resulted in higher ψ_t_ values (Fig 4). In saline soils, 2% intake of the NaCl is used by the plant for osmotically adjust of Na^+^ and Cl^−^in vacuoles [88].

### Ionic stress responses

Tolerance to salinity involves as well important mechanisms for prevention of ion toxicity. This prevention effect might be related to a mechanism of exclusion of toxic ions or their compartmentation. Energy-efficient osmotic adjustment requires compartmentation of Na^+^ and Cl^-^ in vacuoles, and of K^+^ and compatible organic solutes in the cytoplasm [88]. The Na^+^ content in leaves of tolerant populations (DV and BCt) was much lower than in sensitive (DK and BCs), which indicates that persimmon species are able to prevent Na^+^ toxicity. Similar results would be expected in roots; however, the size of the plants did not allow sampling of ions in the roots. To unravel this question, transcriptomic experiments using orthologues of genes described in model plants involved in ion transportation were conducted. The access of Na^+^to the plant vascular system is mediated by non-selective cation channels, but the exclusion from the cell is via a high energy demanding process of Na^+^/H^+^ transporters. In *Arabidopsis*, SOS1, which has antiporter activity, has been demonstrated to play a role in Na^+^ transport outside the cells under saline conditions [35,42]. Therefore, increase of *SOS1* expression should increase salinity tolerance. In persimmon, the transcriptomic study revealed a higher *SOS1* expression in the tolerant *D*. *virginiana* genotypes; however,r this increase of expression may not be related to the salinity treatment. We did not detect significant differences among the BCt and the rest of sensitive populations, which seems to indicate that in these species salt conditions may not trigger the SOS pathway response. In the tolerant plants, with lower content of Na^+^ in leaves, growth was less affected and showed less leaf damage in response to the salinity treatment, whereas they presented higher values of A_CO2_ than the sensitive ones. The hypothesis is that tolerance in persimmon is based on reduction of hydraulic conductance and transpiration to overcome the osmotic stress. This reduction is not damaging the photosynthesis system and affect in a lower scale the plant growth, all together would allow reduction of toxic ions concentration like Na^+^. Another fact supporting this mechanism is that the tolerant DV population showed the ψ_t_ similar in control and saline conditions. The exposition to saline stress in tolerant persimmon is not causing an increase of Na^+^ in leaves or an increase of the osmotic potential.

Regarding to the *HKT1* (high affinity potassium transporter) gene expression, this gene has been linked many times to salinity tolerance in several species, and it is believed that participates in Na^+^ exclusion from the shoot via phloematic transport to the roots [89]. *HKT1* expression prevents Na^+^ accumulation in the higher parts of the plant such as stem and leaves, preventing toxic accumulation on sensible organs. Exposition to salinity environment causes a reduction of gene expression. In persimmon, *HKT1* expression was reduced in saline conditions in all populations. However, the root expression was higher in roots of tolerant plants compared to sensitive which may explain its involvement in tolerance. As *HKT1*-driven tolerance is linked to the tissue-specific expression in other species [90–92], leaf and shoot expression would be necessary for explaining the phenotype of the studied populations.

Regarding to the Cl^-^ exclusion, both in DV and BC_t_ tolerant populations, lower content in Cl^-^ can be associated with the lower relative values of g_s_ and E compared to the control populations. Cl^-^ exclusion has been described as a passive mechanism linked to anion transporters downregulated by ABA [14,93], which also downregulates the stomatal aperture of the plant, limiting the water and ion uptake. Therefore, the reduction on whole-plant transpiration driven by stomatal regulation would contribute to the reduction of Cl^-^ in persimmon tolerant plants. Furthermore, an upregulation on PIP1 and PIP2 aquaporin families has been observed in DV population when exposed to saline conditions. This response has been linked with salinity tolerance in other species [30] as a regulation of the ion imbalance and water flow inside the plant to adapt the ionic and osmotic stresses caused by salinity [94]. The other ion content measured (Ca^2+^, P, Mg^2+^, S) was not affected by salinity treatment.

**Fig 10:**
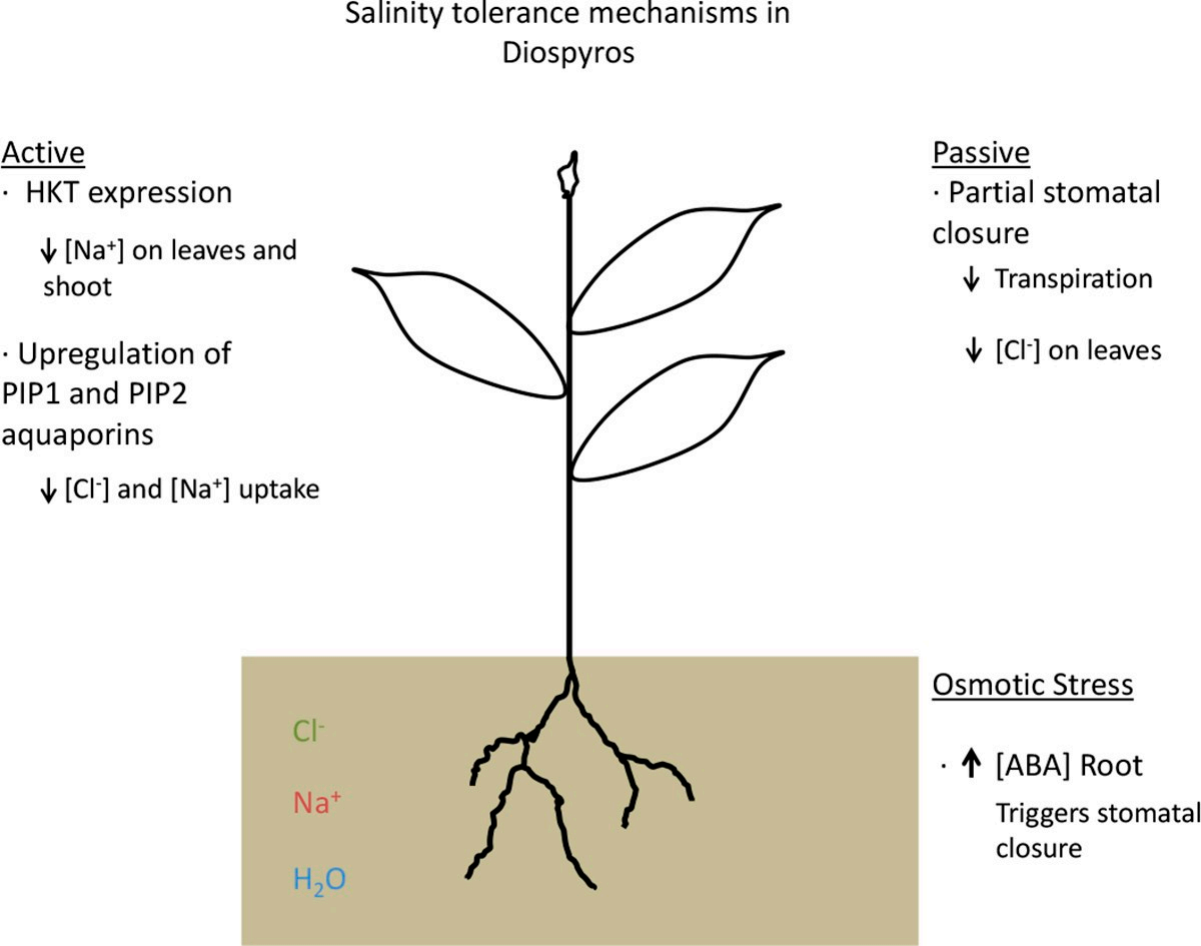
Hypothesis of the salt tolerance mechanisms present in *Diospyros* species. Active mechanisms involved HKT expression and upregulation of PIP1 and PIP 2 aquaporins resulting in a reduction of Na^+^ and Cl^-^ uptake. Passive mechanisms as partial stomatal closure resulted in reduction of transpiration and lower ion accumulation.

In conclusion, persimmon salinity tolerance is based on the reduction of stomatal conductance and decrease of transpiration, preventing the osmotic stress. Besides this mechanism leaf net photosynthesis is higher in tolerant plants and, consequently, growth rate is less affected. The leaf content of toxic ions as Na^+^ and Cl^-^ is also lower in tolerant plants. Necrosis on old leaves for accumulation of toxic ions is associated to sensitive plants. A mechanism of exclusion should be involved. The transcriptomic results do not allow to link expression of *SOS1* to salinity tolerance. The data suggests a potential involvement of *HKT*, however expression data from roots and leaves are required to complete our understanding of the mechanism. Additionally, the upregulation on *PIP1* and *PIP2* aquaporin families detected in tolerant plants exposed to salinity could contribute to regulate the ion imbalance by water flow (Fig 10). Further analysis of the isoforms within PIP families and a persimmon genome assembly would reveal more information.

This is the first approach into the possible mechanism regulating tolerance to salinity of persimmon. Tolerance in hybrids from *D*. *kaki* is now being identified. This fact opens the opportunity of breeding for salinity tolerance and made possible to initiate further studies based on the selected genotypes to further dig into the mechanisms of tolerance to salinity in persimmon species.
