# Correction: A cross population between *D*. *kaki* and *D*. *virginiana* shows high variability for saline tolerance and improved salt stress tolerance

**DOI:** 10.1371/journal.pone.0250193

**Published:** 2021-04-09

**Authors:** Francisco Gil-Muñoz, Juan Gabriel Pérez-Pérez, Ana Quiñones, Amparo Primo-Capella, Jaime Cebolla, Mª Ángeles Forner-Giner, Maria L. Badenes, Mª del Mar Naval

There is an error in affiliation 2 for author Jaime Cebolla. The correct affiliation 2 is: Instituto de Conservación y Mejora de la Agrodiversidad Valenciana (COMAV), Universitat Politècnica de València. Camino de Vera, Valencia, Spain.
